# 106 Preventing a Disastrous Response: Enhancing Preparedness for Burn Mass Casualty Incidents Through Education

**DOI:** 10.1093/jbcr/iraf019.106

**Published:** 2025-04-01

**Authors:** Mark Romero, Natalie Kesler, Tiffany Hockenberry, Karen Richey, Kevin Foster

**Affiliations:** Diane & Bruce Halle Arizona Burn Center; Diane & Bruce Halle Arizona Burn Center; Diane & Bruce Halle Arizona Burn Center; Diane & Bruce Halle Arizona Burn Center; Diane & Bruce Halle Arizona Burn Center

## Abstract

**Introduction:**

The 2023 National Preparedness Report showed an increase in frequency, severity and cost of disasters across the United States from 2020 to 2022. Initial response to a burn mass casualty (BMCI) is the responsibility of the local public health and emergency management agencies who partner will local healthcare organizations. In regions like ours, with a limited number of verified burn centers, it is crucial that staff possess comprehensive burn disaster knowledge and feel confident in their roles in the event of a BMCI. A survey and pre-education test were conducted to identify decreased levels of comfort and potential gaps in knowledge.

**Methods:**

Burn center staff were polled anonymously with a 4-item survey to assess comfort in their role and desire for education. Demographics were polled to help better identify a skillset focal point if targeted education were necessary. The pre-education test was not anonymous and was mandatory for all burn center staff, basic demographics were collected. The 15-item test consisted of 3 subscales: Treatment, Logistics & Operations, and Reporting Instructions. Descriptive statistics were performed.

**Results:**

A total of 82 team members responded to the survey. When asked about understanding of their role in the event of a disaster, 24% reported being uncomfortable and only 12% felt very comfortable. The majority (98%) felt disaster plan education would be beneficial. The knowledge exam was taken by 120 individuals. Respondents performed the best on the 5-item Treatment subscale, with an overall score of 84%. The most frequently missed question was triage location for patients arriving by private vehicle, 55% answered correctly. The overall score for the 7-item Logistics & Operations subscale was 62%. Only 46% of staff were able to identify the designated Incident Commander correctly. The overall score for the 3-item Reporting Instructions subscale was 48%. Only 33% of respondents were able to correctly identify who should report to the ICU.

**Conclusions:**

In the face of a disaster, communication, clear understanding of the chain of command, and each individual’s role are common areas for breakdown in response. While our team demonstrated good knowledge in the treatment subscale, performance in the other two subscales was subpar. We have expanded our internal burn disaster plan to provide additional details for team members, targeting knowledge deficits. Ongoing training and drills are being incorporated so that if disaster strikes, we will be able to respond in a coordinated and effective manner.

**Applicability of Research to Practice:**

This project highlights the need to strengthen disaster education and conduct drills to maintain an acceptable level of readiness for a BMCI.

**Funding for the Study:**

N/A

